# Survey of Barriers and Facilitators to Prescribing Buprenorphine and Clinician Perceptions on the Drug Addiction Treatment Act of 2000 Waiver

**DOI:** 10.1001/jamanetworkopen.2022.12419

**Published:** 2022-05-12

**Authors:** Holly J. Lanham, Jennifer Papac, Daniela I. Olmos, Emily L. Heydemann, Nathalia Simonetti, Susanne Schmidt, Jennifer S. Potter

**Affiliations:** 1 Department of Psychiatry and Behavioral Sciences, University of Texas Health Science Center San Antonio; 2 Department of Population Health Sciences, University of Texas Health Science Center San Antonio

## Abstract

**Importance:**

As opioid-related deaths continue to climb, methods to reduce barriers to prescribing buprenorphine for individuals with opioid use disorder (OUD) are needed. Recent conversations by state and federal authorities targeting low-threshold buprenorphine aim to reduce some barriers to prescribing buprenorphine; however, what remains unclear is whether removal of the requirement to obtain a waiver for prescribing buprenorphine through the Drug Addiction Treatment Act of 2000 (an X-waiver) will be enough to increase access to buprenorphine.

**Objective:**

To assess barriers and facilitators of obtaining an X-waiver and prescribing buprenorphine.

**Design, Setting, and Participants:**

This mixed-method survey study was conducted between September and December 2020; 607 office-based Texas clinicians were surveyed after they attended a buprenorphine X-waiver training course. All attendees between March 2, 2019, and February 28, 2020, were eligible to receive this survey; 126 responses were received (20% response rate: 81 physicians, 37 nurse practitioners, and 8 physician assistants). Data analysis was performed October 2021.

**Main Outcomes and Measures:**

Surveys measured the extent to which clinicians experienced 9 previously identified barriers during the waiver process and in prescribing buprenorphine. The survey included open-ended items assessing facilitating factors to obtaining a waiver and to prescribing buprenorphine for OUD. The barriers were analyzed using χ^2^ tests of homogeneity. Qualitative data were analyzed using a constant comparative method.

**Results:**

Among 126 clinicians who responded, 61 (48.4%) had received an X-waiver; of these waivered clinicians, 22 (36%) were prescribing buprenorphine and 39 (64%) were not. “Complexity of X-waiver process,” “Perceived lack of professional support and referral network,” and “Getting started” were significantly different barriers among waivered and nonwaivered clinicians. Significant differences in barriers experienced between prescribers and nonprescribers were “Getting started” and “Accessing reimbursement for treatment.” The most frequently mentioned facilitators involved changes to the waiver training and the need for networks connecting experienced clinicians with those in the initial stages of readiness for prescribing buprenorphine for OUD.

**Conclusions and Relevance:**

This survey study’s results contribute new understanding of facilitators to obtaining the X-waiver and to prescribing buprenorphine. Furthermore, these findings suggest that to increase access to compassionate evidence-based treatment for OUD, clinicians need ongoing support and mentorship from experienced and knowledgeable clinicians. Interventions aimed at improving access to buprenorphine should focus on facilitating such networks to increase the number of clinicians who obtain an X-waiver and prescribe buprenorphine for OUD.

## Key Points

**Question:**

What are the main barriers and facilitators to obtaining an X-waiver and prescribing buprenorphine in office-based settings after attending an X-waiver training?

**Findings:**

This survey study of 126 clinicians attending a GetWaiveredTX waiver training found that 61 clinicians received an X-waiver; among them, 36% were prescribing buprenorphine and 64% were not. Clinicians noted complexity of X-waiver process, perceived lack of professional support, and referral network as barriers to prescribing buprenorphine; supportive professional networks were a key facilitator.

**Meaning:**

These findings suggest that to increase access to compassionate evidence-based treatment for opioid use disorder, clinicians need ongoing support and mentorship, and interventions to increase the number of clinicians prescribing buprenorphine should focus on facilitating such networks.

## Teaser

This survey study examines barriers to and facilitators of obtaining a waiver to prescribe buprenorphine among office-based clinicians in Texas.

## Introduction

Buprenorphine is effective for patients with opioid use disorder (OUD) and the foundation of best practices for OUD treatment.^1^ During the COVID-19 pandemic, federal regulations increased access to buprenorphine.^2^ Clinicians in office-based settings who complete training to obtain the Drug Addiction Treatment Act of 2000 (DATA 2000) waiver (X-waiver) can prescribe buprenorphine.^3^ Nonetheless, numerous barriers persist to widespread access to medications for opioid use disorder (MOUD) as approximately only 20% to 40% of patients with OUD receive evidence-based medications.^4^

Until recently, physicians needed to complete 8 hours of qualified training and advanced practitioners needed to complete 24 hours before prescribing buprenorphine.^3^ Clinicians then needed to submit their training certificate and a Waiver Notification of Intent to SAMHSA before being able to prescribe buprenorphine in outpatient settings. Not all clinicians will complete the X-waiver process, and clinicians who obtain the waiver often do not prescribe buprenorphine to as many patients as they could.^4^

In April 2021, the US Department of Health and Human Services published new practice guidelines for treating OUD with buprenorphine, substantially reducing the training burden on prescribing physicians. The new guidelines addressed some barriers office-based physicians face in prescribing buprenorphine. Additional barriers remain and less is known about the facilitators that help physicians working in office-based settings initiate and maintain buprenorphine prescribing for patients with OUD.^5^ These include insufficient training and experience, lack of peer support through a clinician’s institution or clinical community, onerous regulatory and reporting requirements, poor reimbursement, and clinician stigma.^2,4,6,7,8,9^ Clinicians also express concerns about their readiness to prescribe buprenorphine, confidently meet the demand for treatment, and adequately serve patients with OUD.^10,11,12^

In Texas, an estimated 1864 opioid overdose deaths were registered in 2020.^13^ Texas has the highest absolute number of people in the country living outside of a 10-mile radius from a waivered clinician.^14^ Texas was also among the top 3 states with the greatest number of individuals with OUD and the lowest number of X-waivered clinicians. To close this gap, statewide initiatives such as the GetWaiveredTX (GWTX) Program, a Texas Health and Human Services–funded initiative at UT Health San Antonio, enabled more than 700 Texas-based clinicians to receive training to prescribe buprenorphine.^15^ GWTX facilitated access to buprenorphine waiver trainings across Texas and supported completion of X-waiver requirements. The intent was to overcome barriers to waivering. In 6 months, GWTX trained 451 waiver-eligible clinicians, 133 (29%) of whom received waivers by 6 months after training.^15^

Underlying the “X the X-waiver” argument (an argument aimed at removing unnecessary barriers to prescribing buprenorphine) is the assumption that doing so will increase buprenorphine prescribing. We conducted this study to better understand key barriers and facilitators to prescribing buprenorphine beyond those related to the X-waiver process. The purpose of this study was to assess the main barriers and facilitators to (1) obtaining an X-waiver and (2) prescribing buprenorphine in office-based settings among clinicians who attended a GWTX training.

## Methods

We distributed an online survey to a cohort of GWTX attendees who received waiver training between March 2019 and February 2020. This follow-up survey was conducted between September 2020 and December 2020 to allow time for clinicians to obtain their waivers and begin prescribing buprenorphine. The survey assessed 9 barriers to office-based buprenorphine prescription previously identified through literature review^15^ using a visual analog scale. Because less is known about facilitators for obtaining an X-wavier and prescribing buprenorphine, we assessed them qualitatively using open text fields. This study was approved by the UT Health San Antonio institutional review board. Patients provided oral informed consent. This study followed the American Association for Public Opinion Research (AAPOR) reporting guideline.^16^

### Study Setting

Approximately 30 million residents of Texas live in 254 counties, only 6 of which are classified as large metropolitan areas. Nearly 1 million Texans need treatment for OUD.^14^ With only 1582 documented X-waivered clinicians in Texas,^14^ rural areas particularly lack access to OUD treatment by waivered clinicians.

### Data Collection

The survey was developed and managed using REDCap at UT Health San Antonio. Research assistants emailed the survey (eAppendix in the Supplement) to 619 waiver-eligible clinicians (DO, MD, PA, or NP) 7 months after the last training. All trainings were conducted in-person just prior to the COVID-19 pandemic. Batches of 50 emails were sent out every week from September to December 2020. The survey distribution permitted adequate time for research assistants to conduct follow-up phone calls with nonrespondents. A reminder email was sent approximately 1 week after the initial email. Research assistants monitored responses and called unresponsive clinicians. Among the 619 clinicians, 12 were unreachable (incorrect email address and no other contact information provided), reducing our sample to 607 clinicians. A $20 Amazon gift card was provided as incentive for study participation.

Survey items were based on a review of peer-reviewed studies (as of February 2019) of barriers experienced by clinicians in obtaining an X-waiver and prescribing buprenorphine. Participants were asked: “Please indicate the extent to which each of the following items has been a barrier for you in obtaining an X-waiver.” By moving a slide bar on a visual analog scale, the answers ranged from “Not [a barrier] at all” (0) to “A great deal” (100). The slider bar was set on the middle of the scale; the scale did not provide an initial score for each barrier, unless participants moved the slider bar. All clinicians, regardless of waiver status, were asked if facilitators did or would have helped in obtaining an X-waiver (“Yes” or “No”). If “Yes,” they were asked to describe the “most helpful facilitating factor.” Clinicians who indicated they had obtained an X-waiver were asked to score the same 9 barriers for prescribing buprenorphine. Waivered clinicians were asked the same question regarding perceived facilitators for prescribing buprenorphine (Figure).

**Figure.**
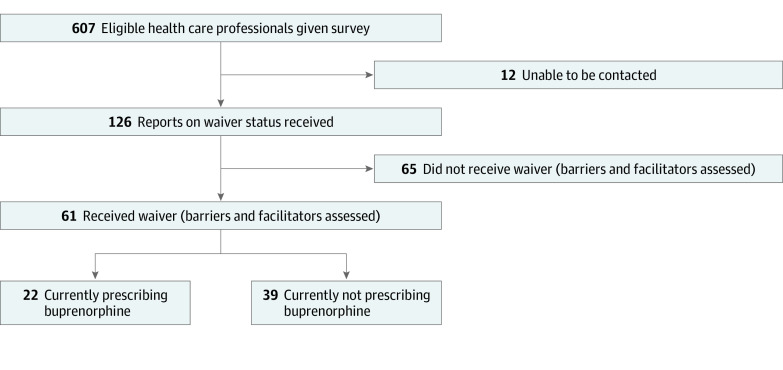
REDCap Survey Decision Tree Based on X-Waiver and Current Buprenorphine Prescribing Status

### Statistical Analysis

#### Quantitative Analyses

We analyzed frequencies for demographic data collected by waiver and prescribing status. Missing scores ranged from 4.8% to 15.9% for barriers. The slider bar of the visual analog scale was first seen by participants at the middle point, or 50, but without an assigned score. Thus, for participants who had moved the slider bar for at least 1 other waiver barrier, we replaced missing scores with the value 50. Participants who moved no slider bars for the waiver barriers were considered nonresponsive, and their scores were coded as missing. The same procedure was followed for buprenorphine-prescribing barriers.

After reviewing the distributions of responses (univariate statistics [eg, mean, median, quartiles] for each score are provided in eTables 1 and 2 in the Supplement), we recoded the barriers by quartiles as follows: “Not at all” (score = 0); “Not a great deal” (score between 1 and 75th percentile of score); and “A great deal” (score equal to or greater than 75th percentile of score). For buprenorphine-prescribing barriers, we merged the “Not at all” and “Not a great deal” categories. These categories were compared across groups based on the clinicians’ waiver and current buprenorphine prescribing status. No a priori hypotheses were formulated. We used χ^2^ tests of homogeneity to determine statistical significance of differences between perception of “Not a great deal” and “A great deal” barriers across groups by waiver and prescribing status.^17^ Statistical analyses were performed using SPSS version 27 (IBM Corp) from February to June 2021. Two-tailed *P* < .05 indicated statistical significance.

#### Qualitative Analyses

These analyses identified themes on facilitators^18^ in 2 phases: (1) content analysis to uncover general themes and develop subcategories under each theme and (2) systematic coding and interpretation of the data. The first phase of analysis followed an open coding approach.^19^ Two researchers coded all text responses. We extracted themes in the data on circumstances clinicians mentioned were, or would have been, helpful to getting waivered and to prescribing buprenorphine. The 2 main coders were a qualitative researcher and a research assistant. When coding medically and practice-oriented text, we included a third team member formally trained in addiction psychiatry. The research team met weekly to discuss coding and preliminary insights from the data. This phase culminated in a list of codes and a codebook with definitions (eTable 3 in the Supplement).

In the second phase, we systematically coded the facilitators data.^18^ One team member independently coded the data, and a second reviewed and provided additional perspective on the coding. Any discrepancies were discussed, and conceptual agreement was reached in weekly full-team meetings. We then analyzed for differences between facilitators described between waivered and nonwaivered clinicians and between waivered clinicians who were or were not prescribing buprenorphine.

## Results

### Clinician Characteristics

Of the 607 office-based Texas clinicians who were surveyed, 126 responded (20% response rate). Among the respondents, 61 (48%) had obtained their X-waiver after attending a GWTX training; of these waivered clinicians, 22 (36%) were prescribing buprenorphine and 39 (64%) were not (eFigure in the Supplement); Most clinicians were MDs and were in primary care, psychiatry, or general acute care (Table 1).

**Table 1.** Survey Participants’ Credentials and Specialties

Characteristic	Participants, No. (%)			*P* value
	Total (N = 126)	Waiver status		
		Nonwaivered (n = 65 [51.6%])	Waivered (n = 61 [48.4%])	
Credentials				
DO	11 (8.7)	6 (9.2)	5 (8.2)	<.001
MD	70 (55.6)	23 (35.4)	47 (77.0)	
NP	37 (29.4)	29 (44.6)	8 (13.1)	
PA	8 (6.3)	7 (10.8)	1 (1.6)	
Specialty				
Addiction specialist	2 (1.6)	2 (3.1)	0	<.001
Primary care	70 (55.6)	44 (67.7)	26 (42.6)	
General acute care	14 (11.1)	5 (7.7)	9 (14.8)	
Psychiatry	26 (20.6)	8 (12.3)	18 (29.5)	
Others	14 (11.1)	6 (9.2)	8 (13.1)	

Abbreviations: DO, doctor of osteopathic medicine; MD, medical doctor; NP, nurse practitioner; PA, physician assistant.

We conducted χ^2^ tests of independence between credential, specialty, and waiver status. All expected cell frequencies were greater than 5. We found a statistically significant association between credentials and waiver status (χ^2^_3_ = 24.64; *P* < .001). Responding physicians were more often waivered than other credentialed clinicians (Table 1). We saw no statistically significant associations between clinicians’ specialty and waiver status.

### Barriers to Obtaining an X-Waiver

Nonwaivered clinicians were more likely to consider “Complexity of [the] X-waiver process” (eg, 22 nonwaivered clinicians [18.5%] answered “A great deal” vs 9 waivered clinicians [7.6%]; *P *= .02), “Perceived lack of professional support and referral network” (21 nonwaivered clinicians [17.6%] answered “A great deal” vs 9 waivered clinicians [7.6%]; *P* = .045), and “Getting started” (eg, 23 nonwaivered clinicians [19.3%] answered “A great deal” vs 10 waivered clinicians [8.4%]; *P* = .03) as larger barriers compared with waivered clinicians (Table 2). Similarly, fewer nonwaivered clinicians reported “Perceived lack of professional support and referral network” and “Getting started” as smaller barriers compared with waivered clinicians. We detected no other statistically significant differences among groups (Table 2). After performing sensitivity analysis and collapsing the “Not at all” and “Not a great deal” categories, the results did not change (eTable 4 in the Supplement).

**Table 2.** Barriers to Obtaining X-Waiver by Clinicians’ Waiver Status

Barriers	Participants, No. (%)			*P* value^a^
	Total (n = 119)	Waiver status		
		Nonwaivered (n = 60 [50.4%])	Waivered (n = 59 [49.6)%]	
Complexity of X-waiver process				
Not at all	23 (19.3)	8 (6.7)	15 (12.6)	.02
Not a great deal	65 (54.6)	30 (25.2)	35 (29.4)	
A great deal	31 (26.1)	22 (18.5)	9 (7.6)	
Understanding DEA regulatory requirements				
Not at all	18 (15.1)	10 (8.4)	8 (6.7)	.79
Not a great deal	67 (56.3)	32 (26.9)	35 (29.4)	
A great deal	34 (28.6)	18 (15.1)	16 (13.4)	
Perceived lack of professional support and referral network				
Not at all	13 (10.9)	6 (5.0)	7 (5.9)	.045
Not a great deal	76 (63.9)	33 (27.7)	43 (36.1)	
A great deal	30 (25.2)	21 (17.6)	9 (7.6)	
Getting started (pharmacy, staff education, practice management)				
Not at all	16 (13.4)	7 (5.9)	9 (7.6)	.03
Not a great deal	70 (58.8)	30 (25.2)	40 (33.6)	
A great deal	33 (27.7)	23 (19.3)	10 (8.4)	
Clinician knowledge for managing patients with OUD				
Not at all	13 (10.9)	8 (6.7)	5 (4.2)	.29
Not a great deal	73 (61.3)	39 (32.8)	34 (28.6)	
A great deal	33 (27.7)	13 (10.9)	20 (16.8)	
Stigma (patient, community, clinician)				
Not at all	24 (20.2)	16 (13.4)	8 (6.7)	.20
Not a great deal	50 (42.0)	23 (19.3)	27 (22.7)	
A great deal	45 (37.8)	21 (17.6)	24 (20.2)	
Managing complex patients and comorbidities (psychiatric and physical)				
Not at all	15 (12.6)	8 (6.7)	7 (5.9)	.67
Not a great deal	74 (62.2)	35 (29.4)	39 (32.8)	
A great deal	30 (25.2)	17 (14.3)	13 (20.9)	
Managing diversion/misuse				
Not at all	9 (7.6)	4 (3.4)	5 (4.2)	.29
Not a great deal	55 (46.2)	24 (20.2)	31 (26.1)	
A great deal	55 (46.2)	32 (26.9)	23 (19.3)	
Accessing reimbursement for treatment				
Not at all	22 (18.5)	10 (8.4)	12 (10.1)	.31
Not a great deal	39 (32.8)	18 (15.1)	21 (17.6)	
A great deal	58 (48.7)	32 (26.9)	26 (21.8)	

Abbreviations: OUD, opioid use disorder; DEA, US Drug Enforcement Administration.

^a^ *P* value from χ^2^ homogeneity tests.

### Barriers to Prescribing Buprenorphine Among Waivered Prescribers

Owing to small sample size, we merged the “Not at all” and “Not a great deal” categories for this χ^2^ analysis. Among waivered clinicians, 14 nonprescribers (38.9%) perceived “Getting started” as a larger barrier compared with clinicians currently prescribing buprenorphine (1 [5.0%]) (*P* = .006). Among nonprescribers, 20 (55.6%) perceived “Accessing reimbursement for treatment” as a larger barrier, significantly higher than among current prescribers (5 [8.9%]; *P* = .03) (Table 3). No other statistically significant differences in proportions were detected.

**Table 3.** Barriers to Prescribing Buprenorphine by Current Buprenorphine Prescribing Status Among Clinicians With X-Waivers

Barriers	Participants, No. (%)			*P* value^a^
	Total (n = 56)	Prescribing status		
		Not prescribing (n = 36 [64.3])	Prescribing (n = 20 [35.7%])	
Complexity of X-waiver process				
Not at all/not a great deal	40 (71.4)	27 (48.2)	13 (23.2)	.43
A great deal	16 (28.6)	9 (16.1)	7 (12.5)	
Understanding DEA regulatory requirements				
Not at all/not a great deal	42 (75.0)	27 (48.2)	15 (26.8)	>.99
A great deal	14 (25.0)	9 (16.1)	5 (8.9)	
Perceived lack of professional support and referral network				
Not at all/not a great deal	42 (75.0)	27 (48.2)	15 (26.8)	>.99
A great deal	14 (25.0)	9 (16.1)	5 (8.9)	
Getting started (pharmacy, staff education, practice management)				
Not at all/not a great deal	41 (73.2)	22 (39.3)	19 (33.9)	.006
A great deal	15 (26.8)	14 (25.0)	1 (1.8)	
Clinician knowledge for managing patients with OUD				
Not at all/not a great deal	42 (75.0)	26 (46.4)	16 (28.6)	.52
A great deal	14 (25.0)	10 (17.9)	4 (7.1)	
Stigma (patient, community, clinician)				
Not at all/not a great deal	37 (66.1)	23 (41.1)	14 (25.0)	.64
A great deal	19 (33.9)	13 (23.2)	6 (10.7)	
Managing complex patients and comorbidities (psychiatric and physical)				
Not at all/not a great deal	41 (73.2)	25 (44.6)	16 (28.6)	.39
A great deal	15 (26.8)	11 (19.6)	4 (7.1)	
Managing diversion/misuse				
Not at all/not a great deal	32 (57.1)	18 (32.1)	14 (25.0)	.15
A great deal	24 (42.9)	18 (32.1)	6 (10.7)	
Accessing reimbursement for treatment				
Not at all/not a great deal	31 (55.4)	16 (28.6)	15 (26.8)	.03
A great deal	25 (44.6)	20 (35.7)	5 (8.9)	

Abbreviations: OUD, opioid use disorder; DEA, US Drug Enforcement Administration.

^a^ *P* value from χ^2^ homogeneity tests.

### Qualitative Patterns

We identified 21 unique facilitators in the data and grouped them into 5 categories based on conceptual relatedness. “Changes to waiver training” and “Provider support and resources” were mentioned most often by participants (Table 4). Under changes to waiver training, “Structural changes to training/process” and “Shorten waiver training time” were mentioned most frequently. Under clinician support and resources, “Education/support after training” and “Increased provider support” were mentioned most frequently. Table 4 lists quotes from respondents for each of these most frequently observed facilitators.

**Table 4.** Highest Frequency Facilitator Codes and Examples From the Data

Codes (frequency)	Examples from data
Education/support after training (12)	“Seeing it in person—being able to not just be in a classroom setting, but actually getting the opportunity to see a physician start/enact the medication process.” “Access to mentors who are already practicing in the area of OUD disorder and treatment.” “Gaining clinical experience for prescribing buprenorphine. Or clinical support from more experience practitioners.” “Shadowing someone in a clinical setting to get more one-on-one assistance with it. I feel like I did the paperwork, but I don’t feel like I have the hands-on knowledge to manage the complexities of it with the waivers, rules, having a psychologist on board, things like that.”
Increase clinician support (8)	“Having mentor can be helpful in obtaining waiver.” “Eliminate the requirements to have an MD sponsor NPs in obtaining the X-waiver.” “I am requiring support and precepting, and it is not due to lack of efforts on your part.” “Don’t really know, I am in just a primary care clinic. My supervising MD had to get certified but does not prescribe the suboxone. We are a rural health clinic so there is really very little professional support for me out there. I only have 4 active cases and have been surprised that I do not have more.”
Structural changes to training/process (8)	“More frequent offerings” (trainings). “Multiple times available for training.” “Having a single public agency/office that is responsible for housing and processing all the X-waiver information, from candidate, training programs, other credentialing processes, to award and renewal of X-waiver with the ‘regular’ individual DEA certificate. The current tri-institution interaction does not add any meaningful security, integrity, or validity to a separate X credentialing, and it most certainly increases the probability of errors, miscommunication, loss of meaningful data and concerns.”
Shorten waiver training time (7)	“Have additional training more readily available for advanced practice registered nurses. Not sure why we have to do so many more hours.” “Make the certification requirements for APNs and PAs less onerous.” “Make the training hours shorter. Even a 2-day training would be better and then online and do an exam.” “Equalizing the number of hours between nurse practitioners and physicians to get certified.”

Abbreviations: APN, advanced practice nurse; MD, medical doctor; NP, nurse practitioner; OUD, opioid use disorder; PA, physician assistant.

Nonwaivered clinicians provided the most data on facilitators for obtaining the X-waiver (eTable 5 in the Supplement). Clinicians who obtained the X-waiver but reported not prescribing buprenorphine provided 15 mentions of facilitators to prescribing. Respondents who had received their waivers and were prescribing buprenorphine for OUD provided the least amount of data.

## Discussion

Understanding barriers and facilitators affecting access to MOUD is an important piece of the large-scale public health effort to reduce opioid-related morbidity and mortality. As OUD incidents have increased, the requirements mandated under DATA 2000 are being reconsidered. In 2021, the US Department of Health and Human Services published updated practice guidelines for treating OUD with buprenorphine, eliminating the 8-hour required training for physicians treating fewer than 30 patients with this medication. Our results support arguments to reform federal policy for buprenorphine-prescribing eligibility.^20^ Despite providing no-cost, convenient waiver training and an online OUD and buprenorphine-focused community of practice (ECHO)^21^ for attendees, less than one-third of GWTX attendees completed the waiver process. Our results suggest the need to fundamentally change the X-waiver requirement and the process for prescribing buprenorphine for patients with OUD. Even with removal of the 8-hour training requirement, barriers related to stigma, support for clinicians in settings with heterogeneous perspectives on OUD treatment, and reimbursement difficulties remain.^5^ Our data suggest the challenges center on the need for professional networks that support supervision and mentorship for new clinicians, and building clinical practice environments where prescribing buprenorphine is accepted.

Many studies have assessed barriers to treating OUD with buprenorphine by broadly assessing the barriers to obtaining an X-waiver and prescribing buprenorphine.^6,12,22^ Before a clinician can prescribe buprenorphine for OUD, several distinct and interwoven activities must occur. Our study systematically assessed barriers to obtaining an X-waiver and prescribing buprenorphine separately, providing additional clarification on the barriers most prominent for each.

Our results found several key differences by comparing respondent perspectives based on their waiver and current prescribing status. Of 126 respondents, 61 were waivered but only 22 were currently prescribing buprenorphine. More nonwaivered clinicians perceived the complexity of the X-waiver process, lack of professional support and referral networks, and getting started as larger barriers compared with waivered clinicians. Fewer nonwaivered clinicians perceived the last 2 barriers as smaller vs waivered clinicians. This makes sense and reflects previous research highlighting challenges regarding the length of X-waiver training and concerns about US Drug Enforcement Administration (DEA) oversight.^23,24,25^

Among our respondents, a significantly higher proportion of nonprescribers considered getting started and accessing MOUD reimbursement as larger barriers vs current prescribers, indicating substantial difficulties in initiating buprenorphine prescribing even after completing X-waiver training. This finding suggests that X-waiver training may only make a subset of eligible prescribers feel comfortable prescribing buprenorphine for OUD. Additional understanding of the specific barriers to initiating buprenorphine prescribing is needed. Similarly, our results demonstrate the uncertainty clinicians face regarding reimbursement for treating patients with buprenorphine.^9,10,11,26^

Our results indicate that getting started is a common substantial barrier for both obtaining an X-waiver and prescribing buprenorphine. This result suggests that clinicians may need well-timed bursts of support or different types of support timed closely with each activity as they work to adopt best practices for OUD treatment. This barrier, along with lack of support and referral networks, highlights the importance of mentorship before, during, and after waiver training. Due to the scarcity of X-waivered clinicians, it is uncommon to gain clinical supervision in most settings unless trainees are highly motivated to do so. Our results are consistent with a prior report suggesting a need for interventions before and after training and focusing interventions with clinicians who a priori agree to provide MOUD services.^27^

Among all respondents, reducing the burden of waiver training and simplifying the waiver process were prominent facilitators. Even with the recently updated practice guidelines, interventions to improve the waiver process should develop methods to streamline the process. Another prominent facilitator across all participants was that a network of support is needed to enable clinicians to successfully adopt best practices for OUD treatment. Nonwaivered clinicians expressed the need for preidentified and active mentorship during training, and the desire to be connected to an experienced clinician as they begin treating patients with buprenorphine. Prescribing clinicians described the need for a connection with 1 missing component in an OUD care network, such as an addiction specialist or pharmacist. Overall, facilitators were more centered on addressing specific aspects of OUD treatment than on getting started.

The nature of facilitators identified in this work provides insight into design and implementation strategies for future interventions. Interventions designed to increase the number of clinicians treating OUD with buprenorphine must acknowledge the complexity of clinicians’ settings.^28^ Interventions that provide training may not be enough. Likewise, barriers to OUD treatment are not static; they are evolving within organizational, community, and political systems.^29^ Interventions to increase the number of clinicians prescribing buprenorphine should build strategies based on local facilitators that reflect the barriers faced in that setting but are easily adaptable.^30,31^

The COVID-19 pandemic’s influence on clinical practice and treatment disruption warrants discussion. Our survey was focused on a set of barriers identified in existing literature at the time and, thus, did not explicitly assess this topic. The open-ended survey items on “other barriers not asked about” had the potential to raise issues related to the pandemic and its impact on care; however, this topic was not prominent in these data. Nonetheless, the shifting landscape of clinical support to telehealth, and overall treatment disruption during the pandemic should be considered in future studies as an important contextual factor for newly waiver-trained clinicians.

### Limitations

Our survey study was cross-sectional and used a single cohort and data collection period. Our response rate was relatively low (20%), which might have influenced results. Presumably, MOUD-engaged clinicians were more willing to participate in our survey. However, responses of waivered and nonwaivered clinicians were similar, suggesting that our sample is representative of GWTX attendees. We conducted this survey prior to the updated practice guidelines eliminating X-waiver training requirement for office-based physicians prescribing to 30 or fewer patients. Additionally, this survey was conducted during the COVID-19 pandemic, which has likely altered office-based OUD treatment practices and impacted perceptions of barriers and facilitators.

## Conclusions

Our study contributes new understanding of facilitators to obtaining the X-waiver and to prescribing buprenorphine. To increase access to compassionate evidence-based treatment for OUD, clinicians need ongoing support and mentorship from experienced and knowledgeable clinicians throughout the X-waiver and prescribing processes. Future interventions aimed at improving access to medications for OUD should focus on facilitating connections and networks of knowledge sharing among experienced clinicians treating patients with OUD and clinicians in the initial stages of readiness for obtaining an X-waiver and prescribing buprenorphine for OUD.

## Notes

Supplement. **eAppendix.** REDCap Survey **eTable 1.** VAS Scores for Waiver Barriers **eTable 2.** VAS Scores for Buprenorphine Prescribing Barriers **eTable 3.** Facilitator Codes with Definitions **eFigure.** DEA X-Waiver and Buprenorphine Prescribing Status Among Survey Participants **eTable 4.** Barriers to Obtaining X-Waiver by Providers’ Waiver Status **eTable 5.** Summary of Facilitators by Waiver Status and Prescribing Status
