# Supplementary material for: Survey of Barriers and Facilitators to Prescribing Buprenorphine and Clinician Perceptions on the Drug Addiction Treatment Act of 2000 Waiver
Source: JAMA Netw Open. 2022 May 12;5(5):e2212419. doi: 10.1001/jamanetworkopen.2022.12419 (PMC9099423; doi:10.1001/jamanetworkopen.2022.12419)
Supplement: Supplement. — eAppendix. REDCap Survey eTable 1. VAS Scores for Waiver Barriers eTable 2. VAS Scores for Buprenorphine Prescribing Barriers eTable 3. Facilitator Codes with Definitions eFigure. DEA X-Waiver and Buprenorphine Prescribing Status Among Survey Participants eTable 4. Barriers to Obtaining X-Waiver by Providers’ Waiver Status eTable 5. Summary of Facilitators by Waiver Status and Prescribing Status [file jamanetwopen-e2212419-s001.pdf]

Supplemental Online Content

Lanham HJ, Papac J, Olmos DI, et al. Survey of barriers and facilitators to prescribing buprenorphine and clinician perceptions on the Drug Addiction Treatment Act of 2000 waiver. *JAMA Netw Open.* 2022;5(5):e2212419.
doi:10.1001/jamanetworkopen.2022.12419

eAppendix. REDCap Survey

eTable 1. VAS Scores for Waiver Barriers

eTable 2. VAS Scores for Buprenorphine Prescribing Barriers

eTable 3. Facilitator Codes with Definitions

eFigure. DEA X-Waiver and Buprenorphine Prescribing Status Among Survey Participants

eTable 4. Barriers to Obtaining X-Waiver by Providers' Waiver Status

eTable 5. Summary of Facilitators by Waiver Status and Prescribing Status

This supplemental material has been provided by the authors to give readers additional information about their work.

© 2022 Lanham HJ et al. *JAMA Network Open.*

eAppendix: REDCap Survey

GetWaiveredTX: Provider Survey

You have been invited to respond to this quick survey on behalf of UT Health San Antonio and of Texas Medication for Opioid Use Disorder (TxMOUD). We're funded by the Texas Health and Human Services Commission. We're interested in learning about your DEA X-waiver process. Whether you are waivered or not, we want to know more about your experience You are being asked to fill out this survey because, according to our database, you completed a GetWaiveredTX training to become eligible to prescribe buprenorphine.

Please take a couple of minutes to fill out this survey. You will be asked to provide your contact information and to briefly tell us about your experiences. If you practice at multiple locations, we would like to know about your unique experience at each of those locations. None of your responses will be reported or published in connection to your name or any contact information you provide.

NOTE: If there are any fields or questions that don't apply to you (e.g., you only practice at one location), please feel free to leave fields or questions unanswered.

Thanks for your time and help!

Contact Information

Name: ____________________________
Credentials (e.g., M.D.): ____________________________
Phone Number: ____________________________
Email: ____________________________

What best describes your primary area of medical practice?
(Select one option from the dropdown menu)

Addiction Medicine
Adolescent Medicine
Critical Care
Emergency Medicine
Family Medicine
General Practice
Geriatric Medicine
Hospice & Palliative Medicine
Hospitalist
Internal Medicine
Obstetrics/Gynecology
Oncology
Psychiatry
Other - Please further specify your primary area of medical practice: ____________________________

DEA X-waiver

Did you obtain a DEA X-waiver after attending a GetWaiveredTX training? (Yes or No)

Please indicate the extent to which each of the following items has been a barrier for you in obtaining a DEA X-waiver:

Complexity of DEA X-waiver process	Not at All (0) - A Great Deal (100)<br>*(Place a mark on the scale above)*
Understanding DEA regulatory requirements	Not at All (0) - A Great Deal (100)

© 2022 Lanham HJ et al. *JAMA Network Open.*

(Place a mark on the scale above)

Perceived lack of professional support and referral network	Not at All (0) - A Great Deal (100)<br>*(Place a mark on the scale above)*
Getting started (pharmacy, staff education, practice management)	Not at All (0) - A Great Deal (100)<br>*(Place a mark on the scale above)*
Provider knowledge for managing patients with OUD	Not at All (0) - A Great Deal (100)<br>*(Place a mark on the scale above)*
Stigma (patient, community, provider)	Not at All (0) - A Great Deal (100)<br>*(Place a mark on the scale above)*
Managing complex patients and comorbidities (psychiatric and physical)	Not at All (0) - A Great Deal (100)<br>*(Place a mark on the scale above)*
Managing diversion/misuse	Not at All (0) - A Great Deal (100)<br>*(Place a mark on the scale above)*
Accessing reimbursement for treatment	Not at All (0) - A Great Deal (100)<br>*(Place a mark on the scale above)*

Other: ___________________________

[If waivered] In order to improve access to evidence-based treatment for Opioid Use Disorder (OUD), we are interested in understanding areas of improvement in relation to the process of obtaining a DEA X-waiver. Do you think there are any circumstances or facilitators that would make the process of obtaining a DEA X-waiver easier? (Yes or No)

[If not waivered] Final Question: In order to improve access to evidence-based treatment for Opioid Use Disorder (OUD), we are interested in understanding areas of improvement in relation to the process of obtaining a DEA X-waiver. Do you think there are any circumstances or facilitators that would make the process of obtaining a DEA X-waiver easier? (Yes or No)

Could you please describe what would be the most helpful facilitating factor to obtain a DEA X-waiver?
____

Buprenorphine

Are you currently prescribing buprenorphine? (Yes or No)

Please indicate the extent to which each of the following items has been a barrier for you to prescribe buprenorphine:

Complexity of DEA X-waiver process	Not at All (0) - A Great Deal (100)<br>*(Place a mark on the scale above)*
Understanding DEA regulatory requirements	Not at All (0) - A Great Deal (100)<br>*(Place a mark on the scale above)*
Perceived lack of professional support and referral network	Not at All (0) - A Great Deal (100)<br>*(Place a mark on the scale above)*
Getting started (pharmacy, staff education, practice management)	Not at All (0) - A Great Deal (100)<br>*(Place a mark on the scale above)*

© 2022 Lanham HJ et al. *JAMA Network Open.*

Provider knowledge for managing patients with OUD	Not at All (0) - A Great Deal (100)<br>*(Place a mark on the scale above)*
Stigma (patient, community, provider)	Not at All (0) - A Great Deal (100)<br>*(Place a mark on the scale above)*
Managing complex patients and comorbidities (psychiatric and physical)	Not at All (0) - A Great Deal (100)<br>*(Place a mark on the scale above)*
Managing diversion/misuse	Not at All (0) - A Great Deal (100)<br>*(Place a mark on the scale above)*
Accessing reimbursement for treatment	Not at All (0) - A Great Deal (100)<br>*(Place a mark on the scale above)*

Other: ___________________________

[If prescribing buprenorphine] In order to improve access to evidence-based treatment for Opioid Use Disorder (OUD), we are interested in understanding areas of improvement in relation to the process of prescribing buprenorphine. Do you think there are any circumstances or facilitators that would make the process of prescribing buprenorphine easier? (Yes or No)

Could you please describe what would be the most helpful facilitating factor to prescribing buprenorphine?
____

[If not prescribing buprenorphine] Final question: In order to improve access to evidence-based treatment for Opioid Use Disorder (OUD), we are interested in understanding areas of improvement in relation to the process of prescribing buprenorphine. Do you think there are any circumstances or facilitators that would make the process of prescribing buprenorphine easier? (Yes or No)

Could you please describe what would be the most helpful facilitating factor to prescribing buprenorphine?
____

[If prescribing buprenorphine] Multiple Locations

Final Question: Do you prescribe buprenorphine at multiple locations? (Yes or No)

Are there any barriers that are specific, or that stand out, at a particular location that get in the way of prescribing buprenorphine? If yes, please describe, and provide the location you are referring to:
____

Are there any circumstances or facilitators that are specific, or that stand out, at a particular location that make it easier for you to prescribe buprenorphine? If yes, please describe, and provide the location you are referring to:
____

© 2022 Lanham HJ et al. *JAMA Network Open.*

eTable 1. VAS Scores for Waiver Barriers.

Barrier	M	Mdn	SD	Minimum	Maximum	Percentiles		
						25	50	75
Complexity of DEA X-waiver process	37.28	30.00	30.79	0	100	10.00	30.00	65.00
Understanding DEA regulatory requirements	39.24	50.00	28.52	0	100	13.00	50.00	60.00
Perceived lack of professional support and referral network	42.08	50.00	30.84	0	100	14.00	50.00	68.00
Getting started (pharmacy, staff education, practice management)	50.82	50.00	31.93	0	100	23.00	50.00	77.00
Provider knowledge for managing patients with OUD	41.45	50.00	27.38	0	100	20.00	50.00	60.00
Stigma (patient, community, provider)	31.91	25.00	28.15	0	99	3.00	25.00	50.00
Managing complex patients and comorbidities (psychiatric and physical)	40.92	45.00	27.74	0	100	19.00	45.00	61.00
Managing diversion/misuse	39.25	40.00	28.35	0	100	15.00	40.00	50.00
Accessing reimbursement for treatment	36.18	45.00	28.58	0	100	8.00	45.00	50.00

© 2022 Lanham HJ et al. *JAMA Network Open.*

eTable 2. VAS Scores for Buprenorphine Prescribing Barriers.

Barrier	M	Mdn	SD	Minimum	Maximum	Percentiles		
						25	50	75
Complexity of DEA X-waiver process	25.64	17.50	26.00	0	90	.25	17.50	50.00
Understanding DEA regulatory requirements	34.27	36.00	27.13	0	90	3.75	36.00	56.75
Perceived lack of professional support and referral network	40.07	46.00	31.35	0	100	3.75	46.00	66.50
Getting started (pharmacy, staff education, practice management)	50.89	63.00	31.45	0	100	21.25	63.00	75.00
Provider knowledge for managing patients with OUD	43.27	50.00	24.84	0	100	20.25	50.00	60.75
Stigma (patient, community, provider)	31.29	26.00	26.19	0	86	5.75	26.00	50.00
Managing complex patients and comorbidities (psychiatric and physical)	39.05	41.50	27.64	0	100	16.25	41.50	60.00
Managing diversion/misuse	34.96	35.00	27.66	0	90	5.75	35.00	50.00
Accessing reimbursement for treatment	34.05	30.00	27.04	0	100	11.00	30.00	50.00

© 2022 Lanham HJ et al. *JAMA Network Open.*

eTable 3: Facilitator Codes with Definitions.

Facilitator Codes (Frequency)	Definitions
**Changes to Waiver Training (41)**	
Structural changes to training/process (8)	Changes to the format for getting the waiver, the structure of the organizations who regulate it, cost, training availability, or the way waiver is granted.
Shorten waiver training time (7)	Reduce time required to complete training (specific to nurse practitioners and physician assistants)
Process materials/summaries (5)	Provide summary of waiver process; reminders to those in progress; summary of dosing information.
Eliminate waiver requirement (5)	Belief that the waiver should not be required (the regular DEA certificate should be sufficient)
Waiver training curriculum changes (4)	Changes/additions to the curriculum during X-waiver training itself.
Technical assistance with waiver process (4)	Provide technical assistance to obtaining the X-waiver.
Mentorship during training (3)	Assign a mentor or study group in the training process.
Streamline/simplify waiver process (3)	Reduce complexity in the X-waiver process.
Clear eligibility guidelines (2)	Provide clear information before/during training on who is eligible to receive/use an X-waiver.
**Provider Support and Resources (20)**	
Education/support after training (12)	Mentor/support/education after of the X-waiver training
Increase provider support (8)	Assistance with obtaining a supervising physician in the provider's clinic/do away with this requirement.
Referral support (2)	Referral support for patients needing MOUD.
**Knowledge and Education (7)**	
Help with staff education (4)	Provide materials to support staff education for MOUD.
Waiver training (2)	Acknowledge the benefit and positive experience in attending waiver training.
Knowledge for managing patients with SUD (1)	Improve providers' knowledge of managing complex patients with SUD.
**Improving MOUD Access (5)**	
Reduce cost of buprenorphine (2)	Reduce the cost of buprenorphine for patients.
Increased access to buprenorphine (1)	Increase the number of pharmacies that prescribe buprenorphine.
Tele-prescription (1)	Allow buprenorphine prescriptions via telemedicine.
Improve access to reimbursement for MOUD (1)	Improve reimbursement for MOUD.
**Managing Stigma (2)**	
Reduce stigma (1)	Reduce the stigma for providers of offering MOUD.
Reduce DEA stigma (1)	Improve providers' perceptions of the DEA/working with their requirements.

© 2022 Lanham HJ et al. *JAMA Network Open.*

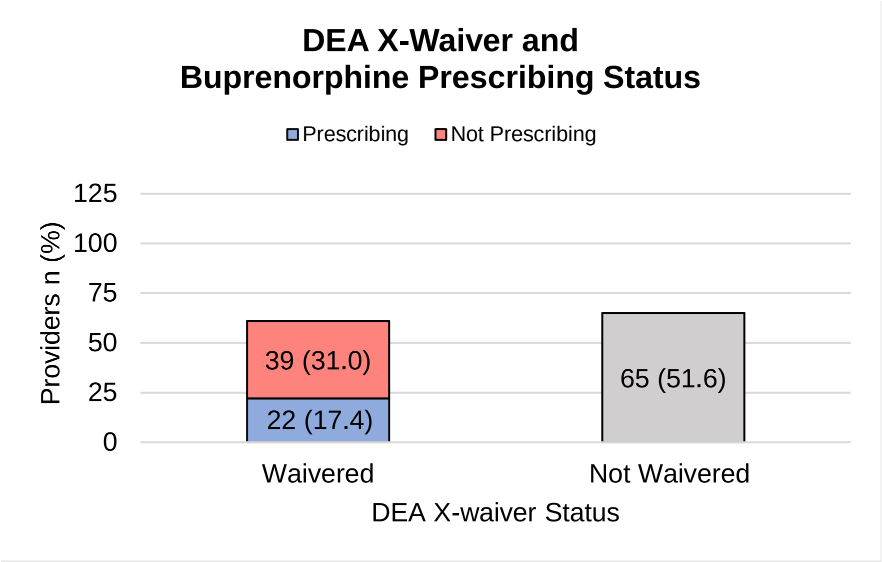

eFigure. DEA X-waiver and buprenorphine prescribing status among survey participants.

© 2022 Lanham HJ et al. *JAMA Network Open.*

eTable 4. Barriers to Obtaining X-waiver by Providers' Waiver Status.

Barriers	Total *119 (100.0)*	Waiver Status *n (%)* Non-Waivered *60 (50.4)*	Waiver Status *n (%)* Waivered *59 (49.6)*	***p*[a]**
**Complexity of X-waiver process**				*.008*
Not at all/Not a great deal	88 (73.9)	38 (31.9)	50 (42.0)	
A great deal	31 (26.1)	22 (18.5)	9 (7.6)	
**Understanding DEA regulatory requirements**				.728
Not at all/Not a great deal	85 (71.4)	42 (35.3)	43 (36.1)	
A great deal	34 (28.6)	18 (15.1)	16 (13.4)	
**Perceived lack of professional support and referral network**				*.013*
Not at all/Not a great deal	89 (74.8)	39 (32.8)	50 (42.0)	
A great deal	30 (25.2)	21 (15.1)	9 (7.6)	
**Getting started (pharmacy, staff education, practice management)**				.009
Not at all/Not a great deal	86 (72.3)	37 (31.1)	49 (41.2)	
A great deal	33 (27.7)	23 (19.3)	10 (8.4)	
**Provider knowledge for managing patients with OUD**				.136
Not at all/Not a great deal	86 (72.3)	47 (39.5)	39 (32.8)	
A great deal	33 (27.7)	13 (10.9)	20 (16.8)	
**Stigma (patient, community, provider)**				.523
Not at all/Not a great deal	74 (62.2)	39 (32.8)	35 (29.4)	
A great deal	45 (37.8)	21 (17.6)	24 (20.2)	
**Managing complex patients and comorbidities (psychiatric and physical)**				.429
Not at all/Not a great deal	89 (74.8)	43 (36.1)	46 (38.7)	
A great deal	30 (25.2)	17 (14.3)	13 (10.9)	
**Managing diversion/misuse**				.116
Not at all/Not a great deal	64 (53.8)	28 (23.5)	36 (30.3)	
A great deal	55 (46.2)	32 (26.9)	23 (19.3)	
**Accessing reimbursement for treatment**				.312
Not at all/Not a great deal	61 (51.3)	28 (23.5)	33 (27.7)	
A great deal	58 (48.7)	32 (26.9)	26 (21.8)	

[a] p-value from Chi-square homogeneity tests.

© 2022 Lanham HJ et al. *JAMA Network Open.*

eTable 5. Summary of Facilitators by Waiver Status and Prescribing Status.

Facilitator Type	Summary
Facilitators to Obtaining X-Waiver (Non-Waivered; n=37)	▪ Access to Active Mentorship<br>– Assign mentors during waiver training, provide opportunities to see an experienced provider start the medication process with an OUD patient, enable knowledge sharing between experienced and non-experienced providers on key topics such as dealing with the DEA, getting started, staff education tools.<br>Example quote: "Preassigning a mentor during training."<br>▪ Physician support of mid-level providers<br>– Nurse practitioners and physician assistants need physician support / supervision in prescribing MOUD.<br>Example quote: "Having a physician who will act as supervising to NP would help me be able to get waivered."<br>▪ Reduce the Burden of Training<br>– Require fewer hours, equalize training hours required for all provider types, provide multiple times available for training, provide more assistance and follow-up after training to help with obtaining waiver, remove the waiver requirement.<br>Example quote: "Get rid of the X-waiver requirement."
Facilitators to Obtaining X-Waiver (Waivered; n=14)	▪ Simplify the Waiver Process<br>– Remove the X-waiver requirement, reduce the number of entities involved in the waiver process, make waiver training free, more clarity on what the waiver is and how to obtain one.<br>Example quote: "I need a one-page description of the process."<br>▪ Provide a Network of Support<br>– Build a MOUD support system for newly waivered providers, mentors needed during training, ongoing education in outpatient and inpatient settings.<br>Example quote: "Ongoing education in outpatient settings."<br>▪ Attend Waiver Training<br>– Engaging waiver training instructors<br>Example quote: "Do the training with Drs. A and B [pseudonyms]! Made it much easier."
Facilitators to Prescribing (Not Prescribing; n=15)	▪ Mentors/experienced prescribers for support and knowledge<br>– Variety of issues non-prescribers mentioned experienced prescriber could help with including tele-prescribing and DEA concerns.<br>Example quote: "Gaining clinical experience for prescribing buprenorphine. Or clinical support from more experienced practitioners."<br>▪ Practice-level Support for MOUD<br>– Practice leadership support for Buprenorphine prescribing, remove limitations on number of providers at given practice who can prescribe buprenorphine, practice staffing to support MOUD.<br>Example quote: "Having a dedicated clinic for this purpose with staff who are knowledgeable on the process and can support."

© 2022 Lanham HJ et al. *JAMA Network Open.*

Facilitator Type	Summary
Facilitators to Prescribing (Prescribing; n=10)	▪ Make It Even Easier<br>– Remove the waiver requirement, make certification requirements for nurse practitioners and physician assistants less onerous, simplify the documentation process.<br>Example quote: “Because buprenorphine is so safe, this should not be a require extra training and licensing to dispense.”<br>▪ Importance of the System of MOUD Care<br>– Need for more referral support, better access to pharmacies stocking buprenorphine, funds for uninsured patients to receive MOUD, and a supporting supervising MD enabling a willingness to treat mindset in non-MD provider.<br>Example quote: “I need increased access to buprenorphine in different pharmacies.”

© 2022 Lanham HJ et al. *JAMA Network Open.*
